# “It’s like I was out there by myself”: The receipt of reentry support among HIV-infected formerly incarcerated individuals in New York City

**DOI:** 10.1186/s40352-020-00108-4

**Published:** 2020-03-10

**Authors:** Tawandra L. Rowell-Cunsolo, Gloria Hu, Rahwa Haile

**Affiliations:** 1 Columbua University, School of Nursing, 560 West 168th Street, New York, NY 10032 USA; 2 Mailman School of Public Health, Columbia University, 722 West 168th St., New York, NY 10032 USA; 3 State University of New York – College at Old Westbury, Natural Sciences Building Room S-208, Old Westbury, NY 11568 USA

**Keywords:** Reentry, HIV, Criminal justice system, HIV care

## Abstract

**Background:**

In the U.S., approximately one in seven HIV-infected individuals experience incarceration at least once in their lifetime. While HIV-infected individuals experience positive health outcomes during periods of incarceration, they tend to experience treatment disruption as they return to their community after custody which results in poor health outcomes. The purpose of this study was to explore the transitional support received from the Department of Corrections during the reentry period.

**Methods:**

We conducted in-depth interviews with 20 HIV-infected formerly incarcerated individuals in New York City. Interviews were audio recorded and transcribed. Three researchers performed line-by-line reading of the transcripts to identify dominant codes and themes that emerged. A mixture of deductive and inductive techniques was used to identify patterns that emerged in the data.

**Results:**

Most of the participants were male and racial and ethnic minorities. There were five dominant themes that emerged during our analysis: 1) variations in the quantity of antiretroviral medication received during transition; 2) linkages to community-based physical health care providers was not well-coordinated; 3) insufficient housing and social resources; 4) structural and social challenges to post-release well-being; and 5) family as a source of resilience.

**Conclusions:**

Discharge support planning should include sufficient medication to prevent treatment disruption and a more comprehensive approach to linkage to community-based healthcare services. Such planning should also include thorough pre-release assessments to identify appropriate levels of support needed, including employment and housing assistance, which will be useful for resource allocation. Broadening public health partnerships may also increase availability and promote accessibility to the most appropriate healthcare services and programs, which may provide better opportunities to receive coordinated care and ensure continuity of care. Finally, ties to family members and other loved ones should be leveraged to help facilitate the achievement of optimal health outcomes among this population.

## Background

Approximately one in seven HIV-infected individuals experience incarceration at least once in their lifetime (Spaulding et al., 2009). It is estimated that 150,000 HIV-infected individuals pass through correctional institutions each year in the United States (Booker et al., 2013). Although they experience optimal antiretroviral (ART) medication adherence and positive health outcomes while incarcerated, due to poor discharge planning and transitional support, the public health benefits gained during incarceration are often lost once individuals return to their home communities (Stephenson et al., 2005; Wohl et al., 2011). Most HIV-infected incarcerated individuals learn of their status during a period of incarceration; up to three-quarters have been diagnosed while incarcerated (Altice, Mostashari, & Friedland, 2001). Hence, many incarcerated individuals have not had the responsibility of managing their condition in a community setting.

Post-incarceration, HIV-infected individuals generally experience treatment disruption and report difficulty maintaining their ART regimen (Baillargeon et al., 2010; Springer et al., 2004; Swan, 2015). Formerly incarcerated HIV-infected individuals are expected to maintain their prescribed regimen in their home community during a period of considerable challenges. They often struggle to manage their condition while attempting to re-establish their lives and pre-incarceration routine (Althoff et al., 2013; Mallik-Kane & Visher, 2008). In fact, in New York City (NYC), being diagnosed with HIV at a city correctional institution is a predictor of delayed medical care (Torian, Wiewel, Liu, Sackoff, & Frieden, 2008). Although the challenges that HIV-infected formerly incarcerated individuals face is increasingly recognized (Draine et al., 2011; Pontali, 2005; Solomon et al., 2014), there is limited research on the support that HIV-infected formerly incarcerated individuals receive from the Department of Corrections as they are transitioning to their home community. Because HIV is a chronic condition that will likely require the lifetime use of antiretroviral therapies to control, such services are essential to facilitating sustained viral suppression post-incarceration. Policies on transition support vary widely on the local, state, and national levels. The importance of identifying potential gaps in services is crucial to developing effective programs designed to improve health outcomes among this vulnerable population.

Existing studies on reentry among HIV-infected individuals focus on challenges including parole restrictions, substance use, social support, and ART adherence (Rich et al., 2013; Travis, 2005; Travis, Solomon, & Waul, 2001). The involvement of the Department of Corrections (DOC) in facilitating services and support post-incarceration is largely overlooked, although most correctional systems seem to report some type of programming related to HIV-related outcomes, including testing, risk behavioral interventions, and linkage to care (Iroh, Mayo, & Nijhawan, 2018). Our study addressed this important gap in the literature by exploring the ways in which formerly incarcerated people living with HIV/AIDS describe the support they received during their reentry into the community.

## Methods

In-depth interviews were conducted with individuals released from prisons and jails in the NYC area. Participants were recruited using convenience sampling. Recruitment flyers were distributed to agencies and non-profit organizations serving the NYC area. Organizations contacted included those specializing in substance use treatment, services for HIV-positive individuals, and services for formerly incarcerated individuals. Potential participants were screened and included if they met the following criteria: (1) at least 18 years of age; (2) released from a correctional institution within the previous five years; and (3) HIV-positive. Interviews were conducted in-person from May 2018 to January 2019, at which point participants were asked to provide their most recent laboratory testing results as proof of HIV status. Participants signed an informed consent form prior to participation. The interviews were conducted by an experienced qualitative researcher in a private room on the Columbia University Irving Medical Center campus and were recorded using a portable recording device. Participants were paid $50 in compensation for their time.

### Measures

We collected quantitative sociodemographic information including age, gender, race, and sexual orientation. Length, history, and dates of incarceration were recorded. HIV ribonucleic acid (RNA) viral load was obtained from participants’ most recent laboratory results.

We used a semi-structured interview guide to qualitatively explore the perceptions of HIV-infected formerly incarcerated individuals regarding the reentry support that they received from the New York State Department of Corrections and Community Supervision (NYSDOCCS). All questions were open-ended to enable us to capture the full range of possible responses; probes were used when appropriate. Questions were focused on the following: 1) the type of services participants received from the NYSDOCCS to prepare for reentry; 2) medical services that they believed were useful; 3) whether participants believed that services received from the NYSDOCCS and community-based agencies were adequate; and 4) challenges experienced when trying to access services in the community.

### Analysis

We used descriptive statistics to summarize the sociodemographic characteristics of the study sample. Digitally recorded interviews were transcribed verbatim using the WReally Transcribe software and manually reviewed by a researcher to ensure accurate transcription. The resulting transcripts and interviewer notes were imported into and coded using NVivo version 12. The analytic research team, consisting of three researchers trained in qualitative methods, performed line-by-line reading of the transcripts to identify emerging themes and assign relevant codes. A mixture of deductive and inductive techniques was used to identify patterns that emerged in the data. First, a priori categories were developed using the interview guide. Second, an inductive approach was used to 1) develop sub-codes based on responses to each interview guide question, and 2) capture participant insights that emerged naturally in the course of the interview but were not covered in the interview guide. The research team met throughout this process and iteratively designed, refined, and finalized the analytic codebook. Using this final codebook, one coder coded all transcripts, and the second coder coded 20 percent (*n* = 4) of interviews to ensure inter-rater reliability. A summary outlining major themes across interviews was produced. All quantitative data were analyzed using The Statistical Package for the Social Sciences (SPSS) version 26.

## Results

There were 20 participants in the study. Participant characteristics are listed in Table 1. Participants were predominantly male (80%), identified as people of color (95%), and heterosexual (80%). Participants had been residing in the community for an average of 1.59 years (SD = 1.36). Our analysis led to the identification of five dominant themes that emerged in participation explanations of their post-release transition: 1) variations in the quantity of post-release medication received; 2) linkages to community-based physical health care providers was not well-coordinated; 3) insufficient housing and social resources; 4) social and structural challenges to post-release well-being; and 5) family as a source of resilience.

**Table 1:** Socio-demographic characteristics

Attribute	N (%)
Sex	
Male	16 (80%)
Female	4 (20%)
Sexual orientation	
Heterosexual	16 (80%)
Lesbian/Gay/Bisexual/Transgender	4 (20%)
Race	
White	1 (5%)
Black	15 (75%)
Hispanic/Latino	4 (20%)
Marital status	
Single	10 (50%)
Married	2 (10%)
Other	8 (40%)
Education	
Less than HS	8 (40%)
GED	9 (45%)
HS or higher	3 (15%)
Housing	
Considers self homeless	15 (75%)
Housing situation	
Own house or apartment	6 (30%)
Rooming/boarding house	13 (65%)
Shelter or welfare boarding house	1 (5%)
Has health insurance	20 (100%)
Viral load	
Undetectable	6 (30%)
Average (among detectable)	16,123.07
Average time since release (years)	1.59 (1.36)
Average length of most recent incarceration (years)	4.51 (SD = 4.75)

### Variations in the quantity of ART medication received during transition

To remain virally suppressed, ART medication is needed daily. The amount of medication received from the NYSDOCCS while they were being discharged largely varied among participants. While some (25%) participants reported having received at least a thirty-day supply of ART medication upon release, others (35%) reported receiving less than a month's supply, which they experienced as profoundly insufficient to support their health during the post-release transition. Participant 16 reported that they received “nothing, zero, squat” during this period. Similarly, participant 11 reported that the DOC released them with “no medication or nothing at all… it was rough when I first came out as far as my medication. And I really needed it. And it’s like, you know, when you come home, you go to parole, and like--- nobody really wants to help you. It’s like I was out there by myself.” Participant 3 similarly described feeling medically unprepared upon release, as though she was left to fend for herself. The participant stated that “they gave me a week’s worth of HIV meds… and from there on, just told me to find out where the nearest HIV clinic was.” Participant 15 characterized post-release medication distribution as entirely haphazard, stating that the NYSDOCCS didn’t “give me any medication to go home with. The medication that I had on me, I took with me… they don’t give you no supply. It’s whatever you got is what you go with. So if we have one bottle left… that has two more pills in it, that’s what you’re going home with.”

### Linkages to community-based physical health care providers was not well-coordinated

Some participants described having met with representatives from community-based agencies before they were discharged. During these meetings, these representatives would discuss the participant’s post-release plans and medical services that the participant is interested in or eligible to receive. Although some participants described these meetings as helpful in exposing them to potential post-incarceration services, many participants reported that they needed more support to locate a community-based provider.

Twenty-five percent of participants reported having received insufficient support in connecting to physical health providers upon release. For example, participant 3 described the immense stress associated with feeling that they were alone in navigating their post-release medical care. They stated that “it would have been helpful if… they would have set up an appointment with a doctor before I got out—that would have been much less stressful… basically I had to do all the footwork myself.” Participant 10 also articulated feeling alone in navigating the complex process of identifying a provider post-release, stating “I had to go around myself and basically find one, you know. That should already be in place when you walk out… already have a provider established.” Participant 11 also reported that he had “problems with” scheduling medical appointments upon release but that he luckily was eventually able to locate his pre-incarceration primary care provider. Participant 12 described struggling to gain consistent medical care post-release because he did not have health insurance. He explained that upon release he “didn’t have Medicaid or anything. I couldn’t get no medicine. I would go to the emergency rooms and stuff like that…. It was very difficult for me to get meds.”

However, some participants (20%) described feeling that the NYSDOCCS connected them well with post-release physical healthcare in the community. For example, participant 13 reported that he was able to get an appointment with his healthcare provider “right away” post-release. Similarly, participant 17 reported that he easily scheduled a physical health appointment upon release. The most helpful services were reportedly those that offered comprehensive healthcare at one location. Because participants reported a range of comorbidities, including cardiovascular disease and psychiatric conditions, they appreciated having the ability to visit more than one provider at a single location. Some agencies provided case management services; being assigned a case manager to assist with locating the proper services resulted in a smoother transition to the community. According to participants:

“Well the case manager helped me a lot, you know, [inaudible] me through like step by step. Be a lot of help for - for me.” – Participant #9, Female, 53.

“Well my medical services I’m receiving, I get everything- it’s at [location]. I have my primary care physician there, … And I also have my psychologist there… and my psychiatrist. And they all have, you know been great, you know, helping me in me navigate through this, uh, place called society [laughs]. But yes, it’s been great because every - all my providers is in the same building, which is really great too, you know, my primary, my psych and my psychiatrist. And they’ve been very good, very helpful. Very helpful.” – Participant 12, Male, 55.

### Insufficient housing and social resources

Despite having a strong desire for a productive reentry into the community, participants experienced many barriers to actualizing this. In particular, they described insufficient linkages to housing and other social resources, as well as multiple forms of discrimination in housing and employment.

Many (45%) participants reported feeling dissatisfied with their post-release housing and other social resources. In particular, they reported struggling to secure safe and stable housing. Participant 17 explained that “it was hard for me to get housing because I didn’t have all my paperwork, and I didn’t know where to get it, and I don’t know who to ask,” and suggested that the NYSDOCCS should better link people to housing pre-release, proposing that “maybe they could hook you up with… HASA [HIV/AIDS Services Administration] before you come home” so that the struggle for stable housing would be less overwhelming. Similarly, participant 2 described wanting additional housing-related support, suggesting that the NYSDOCCS could provide “more monitoring to see if you got a place to go.”

Others reported experiencing multiple sources of stigma and discrimination, which prevented them from securing stable housing. Participant 2 explained that housing discrimination was ubiquitous as “people don’t want to take the vouchers from HASA. You know, they find out you got money from HASA… that’s a special program for people with the virus… they don’t want to accept the vouchers.” Participant 11 also reported being denied housing because of the stigma associated with their HIV status, and that while trying to secure an apartment he was told “Yea um, you said programs? What kind of program you got? HASA? Nah, I’m not, we don’t accept, we don’t accept that—no we don’t accept programs.” Participant 11 also reported being personally denied housing because of stigma and discrimination related to their past criminal justice involvement. He was told that he had “too many felonies,” and explained that this “really sent me for a loop… I broke down in the office. I started crying. I’m like, y’all don’t want to hear nothing. Well if that’s the case then ... just send me back to jail…. I can’t get rid of the felonies I got - they with me until I leave.” Despite their desire to move forward, how others interpret the immutable fact of their past criminal justice involvement continues to bar them from a better life.

Participants also described wanting more basic transitional support to help ease their post-release transition. For example, participant 14 described the need for food immediately post-release, “because it’s a lot of people that I know that - especially me, I was starving.” Participant 5 similarly described needing assistance navigating complex institutional barriers to accessing social benefits because “all my services from food stamps services to Social Security benefits, they closed everything so when I came out I had nothing.” Similarly, participant 9 described that she had to wait almost two months to enroll in an outpatient drug treatment program; she felt that it would have helped her to “go straight into a program.” Others discussed wanting to have post-release access to skills-building programs to help them gain employment and financial stability. Participant 2 described wanting “a little more … assistance to set him up with jobs, or schooling… to be self-sufficient,” and participant 13 similarly described wanting to be involved in “some kind of training program… like to help me get a job… I really want to become a nurse.” Participants desired a stable and productive life in the wider community, yet they described insufficient housing and social resources to help them build a successful transition.

### Structural and social challenges to post-release well-being

Participants (30%) also described multiple structural and social challenges to their health and well-being post-release. Participant 10 noted that managing the constraints of employment made it difficult for them to remain drug adherent. He explained that he was “undetectable for over fifteen years, and … got a job so through the hustlin’ bustlin’ of getting back into the workforce, I missed quite a few days of medication and my viral load went from undetectable to 900.” In contrast, participant 13 described his fear of discrimination in pursuing employment, stating that “One of the challenges is feeling discriminated against in reference to … trying to get a job, like working in a hospital… because that’s mainly what I wanted to do. But I feel that due to my HIV status and my criminal background, you know, I might, you know, see some kind of discrimination.” Participants’ ongoing struggles with addiction also pose a major challenge to their successful post-release transition. Participant 17 for example explained that “The biggest challenge with me was not picking up heroin again.” Intensive, immediate, and ongoing post-release support in the areas of medication adherence strategies, coping with discrimination, and maintaining sobriety may greatly help formerly incarcerated people living with HIV/AIDS to actualize their plans and hopes for post-release life.

### Family as a source of resilience

Despite the many challenges that they navigate immediately post-incarceration, many (30%) participants also described family and other loved ones as important sources of strength and resilience. Participant 4 explains that “when I really need some motivation towards taking my medication, I talk to my son and my grandsons.” Other participants explained that loved ones help them to remember to see their clinicians and to adhere to their medication regimen. Participant 9 explained that “my husband—he’s my man of heaven… he’ll tell you, did you schedule your appointment,” and that her grandkids say “‘Grandma take your medication’—he don’t know what kind of medication it is, but he says ‘Grandma, your medication. I don’t want you to be sick Grandma.’” Participant 11 described that “my brother calls me sometime in the morning like five o’clock, six o’clock, to remind me to take my medication.”

## Discussion

Our findings indicate that formerly incarcerated HIV-infected individuals in NYC receive inconsistent levels of support during their transition from custodial settings to the community. In our study, we were able to characterize the types of support that they received while they were returning to the community, including antiretroviral medication, linkage to community-based healthcare providers, and assistance securing post-incarceration housing. Participants experienced other obstacles, including social challenges such as dim employment prospects and substance use that typically reduce the likelihood of a successful reintegration into the community (Holzer, Raphael, & Stoll, 2003; Travis, 2005; Travis et al., 2001). However, they were encouraged by supportive family members who encouraged them to be remain adherent to their medication regimen.

While most participants reported receiving antiretroviral medication prior to being released, the amount of medication they received varied greatly. Previous research has demonstrated that only a small percentage of HIV-infected individuals are able to access enough ART medication to avoid treatment disruption post-incarceration (Baillargeon et al., 2009). Receiving ART medication during the reentry period is critical because at least 90 percent adherence to ART is required for sustained HIV replication suppression (Liu et al., 2006; Raffa et al., 2008). While further research is needed to determine what might constitute a sufficient amount of medication to provide upon release, barriers which include discontinuity in healthcare providers and competing demands for other necessities (housing, food, etc.) must be taken into account.

Our findings suggest the linkage to care is inconsistent, which was also demonstrated in a study on prisoners returning to communities in Connecticut (Loeliger et al., 2018). While some participants were satisfied with the level of support they received, others expressed a need for additional support and experienced frustration upon learning that they were responsible for locating a community-based provider. Representatives from agencies that visit correctional institutions to develop referrals to community-based agencies should clearly communicate the types of services and support that are available to HIV-infected individuals post-incarceration. Since inadequate post-release support is associated with engagement in high risk behaviors (Luther, Reichert, Holloway, Roth, & Aalsma, 2011), performing thorough pre-release assessments is an important step in informing appropriate discharge support and reducing transmission potential.

More extensive linkage to care services may be needed to provide adequate levels of support for a population that routinely experiences treatment disruption (Baillargeon et al., 2010; Fox et al., 2014; Haley et al., 2014; Harding, Morenoff, & Herbert, 2013). Since linkage to a community-based provider is crucial to promoting treatment engagement, it may be useful for the DOC to pursue additional community-based partnerships and better care coordination to ensure that HIV-infected formerly incarcerated individuals have access to the full-range of HIV primary care services and relevant programming during this vulnerable period. Because they primarily rely on government assistance for basic necessities post-incarceration (Hallett, 2012; Richards & Jones, 1997), the role of the DOC in facilitating enrollment in relevant health-based programs may result in a smoother transition and a more robust improvement in health outcomes.

Although participants received housing assistance from a government assistance program, HASA, they had trouble securing permanent housing, which is typical for formerly incarcerated individuals and HIV-infected individuals (Aidala et al., 2016; Pleggenkuhle, Huebner, & Kras, 2016). Due to restricted access to public housing and other forms of housing assistance, formerly incarcerated individuals typically experience residential instability and/or chronic homelessness (Harding et al., 2013; Metraux & Culhane, 2004). Unstable housing is associated with poor HIV treatment outcomes among HIV-infected formerly incarcerated individuals, which underscores the importance of providing critical resources to address the lack of affordable housing options for this population (Zelenev et al., 2013). While participants expressed gratitude for housing assistance, they also reported that government assistance was stigmatized, and some landlords refused to accept HASA rental assistance vouchers. Landlord refusal of housing vouchers has been previously documented (Cunningham et al., 2018) and further limited participants’ housing options.

Previous research suggests that with few housing options, formerly incarcerated individuals may subsequently resettle in environments where they are routinely exposed to drugs (Mkuu, Rowell-Cunsolo, & Harvey, 2019; Travis, 2005), complicating efforts to promote substance use treatment and recovery efforts for this population. Because of this risk and the recognition that housing support is a central component of successful reentry (Lutze, Rosky, & Hamilton, 2014), targeted programs that prioritize placement in substance-free residential communities and those that include resources on behavioral health treatment and services may be meaningful for this population. Our findings are also consistent with previous studies that have identified difficulty finding employment as a primary challenge of reentry (Baer et al., 2006; Stafford, 2006). Employment training programs have shown promise in improving post-incarceration employment outcomes and should be considered in preparing prisoners for reentry (Duwe, 2015).

Positive reinforcement from family members provided encouragement and helped participants gain greater awareness of the importance of managing their condition. Facilitating closer contact with loved ones prior to release, and explicitly supporting, empowering, and building upon these connections during the immediate post-release period may improve their health and social outcomes. Because relationships with family and friends are impacted during periods of incarceration (Anderson-Facile, 2009; Baer et al., 2006; Naser & La Vigne, 2006), interventions that foster engagement and ongoing communication with loved ones may help compensate for deficiencies in transitional support programs (Berg & Huebner, 2011; Brunton-Smith & McCarthy, 2017).

### Limitations

Although this study has public health significance, it does have limitations. The small sample size impedes our ability to draw broad conclusions based on the study findings. The participants were also drawn from a single large urban area using convenience sampling. Hence, their experiences may not be representative of those who were released from other prison systems in other geographical areas. Despite these limitations, we believe that the findings provide some guidance on potential areas to target to improve the reentry experiences of HIV-infected individuals.

## Conclusions

In conclusion, our findings suggest that HIV-infected individuals transitioning from custody to the community experience inconsistencies in support from the DOC. Discharge support planning should include sufficient medication (or enough medication to prevent disruption) and a more comprehensive approach to linkage to community-based healthcare services. Such planning should include thorough pre-release assessments to identify appropriate levels of support needed, which will be useful for resource allocation. Broadening public health partnerships may increase availability and promote accessibility to the most appropriate healthcare services and programs, which may provide better opportunities to receive coordinated care and ensure continuity of care. Assistance with obtaining more permanent, secure housing should be incorporated into reentry planning programs for HIV-infected individuals, as it is essential to promoting successful reentry into the community (Teixeira, Jordan, Zaller, Shah, & Venters, 2015). Finally, ties to family members and other loved ones should be leveraged to help facilitate the achievement of optimal health outcomes among this population.

## Data Availability

Because participants provided personal information about their background and personal behavior, the data are not publicly available.

## Abbreviations

ART: Antiretroviral
DOC: Department of Corrections
HASA: HIV/AIDS Services Administration
HIV: Human Immunodeficiency Virus
RNA: Ribonucleic acid
SRO: Single room occupancy
